# Genetic basis of benzimidazole resistance in *Teladorsagia circumcincta* in Ireland

**DOI:** 10.1186/s13620-017-0087-8

**Published:** 2017-02-13

**Authors:** Jason D. Keegan, Barbara Good, Theo de Waal, June Fanning, Orla M. Keane

**Affiliations:** 1 Animal and Bioscience Department, Teagasc, Grange, Dunsany, Co. Meath, Ireland; 2 Animal & Bioscience Dept, Teagasc, Mellows Campus, Athenry, Co. Galway, Ireland; 3 School of Veterinary Medicine, University College Dublin, Belfield, Dublin 4, Ireland; 4 Department of Agriculture Food and the Marine, Central Veterinary Research Laboratory, Backweston, Co. Kildare, Ireland

**Keywords:** Sheep, Anthelmintic, Resistance, Benzimidazole, Teladorsagia, β-tubulin

## Abstract

Resistance to benzimidazole (BZ) anthelmintics is common in ovine nematodes of economic importance. Single nucleotide polymorphisms (SNP) at three positions in the isotype 1 β– tubulin gene have been associated with BZ resistance and molecular tests for the detection of BZ resistance have been developed. In order to determine if such tests are practicable in Ireland the polymorphisms associated with BZ resistance must be identified. To this end, BZ-resistant nematodes were recovered from four farms in Ireland. Resistant *Teladorsagia circumcincta*, *Cooperia curticei* and *Trichostrongylus colubriformis* were recovered, with resistant *T. circumcincta* the most common and the only species studied further. Sequencing of the isotype 1 β–tubulin gene from resistant *T. circumcincta* identified a T - A transition, resulting in an F200Y substitution known to be responsible for BZ-resistance, on three of the farms. However, on the fourth farm the frequency of the resistant A allele was only 0.33 indicating another BZ resistance mechanism may be present on this farm. An additional polymorphism resulting in a substitution of glutamate for leucine (E198L) was also found on this farm at low frequency (0.17). No polymorphisms at position 167 were identified on any farm. Therefore, molecular tests to detect BZ resistance in *T. circumcincta* in Ireland could prove useful; however, they may result in some instances of resistance remaining undetected.

## Introduction

Anthelmintic drugs are heavily relied upon to control the effects of parasitic helminths in sheep, but their overuse has led to the development of anthelmintic resistance (AR). Resistance to benzimidazole (BZ) on Irish sheep farms has been reported [1–3] but the prevalence may be underestimated, given the insensitivity of diagnosing AR based on faecal egg count [4]. In BZ-susceptible nematodes, BZ binds to tubulin disrupting the formation of microtubules, resulting in a reduction in glucose uptake and protein secretion, which leads to starvation and death. In resistant nematodes, an amino acid change in the tubulin protein prevents BZ from binding [5]. Three non-synonymous single nucleotide polymorphisms (SNP) in the isotype 1 β-tubulin gene have been associated with BZ resistance in a variety of ovine gastrointestinal nematode (GIN) species. The most common of these polymorphisms results in a phenylalanine to tyrosine substitution at position 200 (F200Y), while the other SNP result in a phenylalanine to tyrosine substitution at position 167 (F167Y) or a glutamic acid to alanine substitution at position 198 (E198A) [6–8]. However, BZ resistant nematodes have been found without any of the three known mutations, suggesting the existence of additional determinants of resistance [9]. The polymorphisms responsible for BZ resistance in Irish ovine nematodes remain unknown.

Recently molecular tests for the detection of BZ resistance in a variety of ovine nematode species have been developed [7, 10, 11]. The objective of this study was to investigate the molecular basis for BZ resistance in Irish field populations of *Teladorsagia circumcincta*, one of the most common GIN species found in sheep in Ireland [12, 13], with a view to establishing whether such molecular methods of BZ-resistance diagnosis are practicable in Ireland.

## Materials and methods

### Nematode isolation

Four geographically dispersed farms (Wicklow, Kerry, Roscommon and Galway) with a confirmed history of BZ resistance were recruited [1]. Four lambs were purchased from each farm and brought to the Department of Agriculture, Food and the Marine (DAFM) farm in Co. Kildare. The lambs were housed in quarantine with straw bedding as required. Lambs were fed hay and a commercial pelleted ration with free access to water. Lambs were given 2 days to acclimatise before faecal samples were collected per rectum and faecal egg count (FEC) determined using the modified McMaster method with a minimum sensitivity of 25 eggs per gram (epg) [14]. Lambs were subsequently treated with a BZ product (Systamex®, 5 mg/kg per os (p.o.), Schering Plough) as per manufacturer’s instructions. Fourteen days post-treatment a faecal samples was collected and FEC determined and lambs were treated a second time with the same anthelmintic product at the same dose rate. Ten days (farms A and B) or 13 days (farms C and D) after the second BZ treatment, faecal samples were collected and FEC determined as described above in order to calculate the reduction in egg count following the second treatment. The lambs were euthanised 14–16 days after the second BZ treatment and the gastrointestinal tract removed. The abomasum was processed to recover GIN according to the method previously described [15] with the exception that GIN were stored in water after washing; while the first 10 m of the small intestine was processed to recover GIN as described previously [16]. Adult male nematodes were speciated based on their morphological features [14] and live worms were preserved individually in RNAlater or PBS and frozen at −20 °C. For each farm, faecal egg count reductions were calculated as ((mean FEC pre-treatment – mean FEC post 2nd treatment)/mean FEC pre-treatment)*100.

### DNA extraction and isotype 1 β–tubulin gene sequencing

DNA was extracted from individual adult male *T. circumcincta* using the Qiagen QIAmp® DNA micro kit as described in the manufacturer’s protocol. DNA was eluted in 15 μL of nuclease free water and preserved at −20 °C. Initially the full 4216 bp isotype 1 β–tubulin gene was amplified in a reaction containing 0.5 μM Tcirc_btubulinF and Tcirc_btubulinR primers (Table 1), 200 μM dNTPs, 1 × High Fidelity buffer, 1 unit of Phusion high fidelity DNA polymerase (New England Biolabs, UK) and 1.5 μL of template DNA in a 50 μL reaction. The reaction was denatured at 98 °C for 2 min followed by 30 cycles of 98 °C for 10 s; 64.9 °C for 10 s and 72 °C for 2 min with a final extension step for 72 °C for 5 min. Subsequently another PCR was designed to amplify the β-tubulin gene in two ~2 kb fragments (Tcirc_btubulinF and Tcirc_btubulin_intR; Tcirc_btubulin_intF and Tcirc_btubulinR; Table 1), using the same conditions as above except with an elongation time of 1 min. Cloning of the 4216 bp PCR product was also conducted for 8 nematodes as direct sequencing of the PCR products was found to be unreliable. The PCR product was gel purified using the QIAquick PCR purification kit (Qiagen, Germany) and cloned using the TOPO XL cloning kit as per manufacturer’s instructions. Between 1 and 4 plasmid inserts per individual nematode were sequenced. The amplification and sequencing primers used are listed in Table 1. Purified PCR products or plasmids were sent for Sanger sequencing at Source Bioscience (Waterford, Ireland) or Beckman Coulter Genomics (Essex, UK). Six sequencing primers were used to obtain the entire sequence including the introns. Sequences were assembled in bioedit using cap3 sequence assembly software [17] to generate the complete β-tubulin sequence per individual. Sequences were aligned in MEGA 5 using MUSCLE [18] and compared to the *T. circumcincta* mRNA for β-tubulin sequence (GenBank ID: Z69258.1) in order to identify coding sequence SNP.

**Table 1:** Primer sequences for *Teladorsagia circumcincta* isotype 1 β–tubulin amplification and sequencing

Primer Name	Function	Sequence 5′ - 3′
Tcirc_btubulinF	amplification	CCTCGACTACAATCATGCGTG
Tcirc_btubulinR	amplification	CGCAACCAATGTGTATTTCG
Tcirc_btubulin_intF	amplification	GGACAACTTTTCCGTCCAGA
Tcirc_btubulin_intR	amplification	CAACCCTCTGCCTCTTTACG
TCSQ1R	sequencing	AGCCAGGACAGAGAACAACG
TCSQ2F	sequencing	AGTCCAGGAGAGGGGAAAAA
TCSQ3F	sequencing	AATGCGAAAGTGCTTCACCT
TCSQ4R	sequencing	AACTGTCCAGGGAATCGAAG
TCSQ5F	sequencing	TGCTTCCGCACCTTAAAACT
TCSQ6F	sequencing	CCCACGACTGCTTTGTGTAA

## Results

Faecal egg count reductions calculated from four lambs from each farm indicated BZ resistance on each farm with nematodes surviving two rounds of BZ treatment. BZ resistant *T. circumcincta* was recovered post-mortem from lambs from all four farms and was the only species present post-treatment on all farms tested. *C. curticei* was recovered post-mortem from lambs from three farms while *T. colubriformis* was recovered from lambs from two farms (Table 2). Only *T. circumcincta* was studied further.

**Table 2:** Faecal egg count, species surviving benzimidazole treatment (s) and substitution frequencies at positions 198 and 200 of the β –tubulin protein for each of the farms

	Faecal egg count^a^				Species recovered post-mortem			Substitution frequency	
Farm	Pre-treatment (epg)	Post 1^st^ treatment (epg)	Post 2^nd^ treatment (epg)	% Reduction^b^	*Teladorsagia circumcincta*	*Cooperia curticei*	*Trichostrongylus colubriformis*	E198L	F200Y
Wicklow	1370	568	794	42	+	+	-	0.17	0.33
Kerry	889	409	406	54	+	+	-	-	1
Roscommon	1050	647	469	55	+	+	+	-	1
Galway	1122	263	194	83	+	-	+	-	0.94

^a^represents the average of 4 lambs per farm

^b^reduction calculated after 2 benzimidazole treatments

The full sequence for the isotype 1 β-tubulin gene was obtained for 36 individual male *T. circumcincta* (Wicklow *n* = 9; Galway *n* = 8; Roscommon *n* = 7; and Kerry *n* = 12). The most common polymorphism was a T to A transition (T**T**C – T**A**C) resulting in a phenylalanine to tyrosine substitution at codon 200 (F200Y). This polymorphism had an overall frequency of 0.79 among the worms which survived two rounds of BZ treatment. However, the frequency differed between the farms, with the polymorphism fixed in the worms from Kerry and Roscommon, with all individuals TAC/TAC homozygotes. On the Galway farm, 7 of the surviving nematodes were homozygous for the polymorphism with the remaining individual heterozygous (frequency = 0.94). However, on the Wicklow farm the frequency of TAC was only 0.33, with only one *T. circumcincta* which survived BZ treatment homozygous for the polymorphism. A further four surviving individuals were heterozygous while the remaining four individuals had the BZ susceptible genotype (TTC). Interestingly, two nematodes from the farm in Wicklow encoded non-synonymous substitutions at position 198 resulting in a substitution of glutamate for leucine (E198L). One of these nematodes was homozygous for the TTC BZ susceptible allele at position 200, with the other heterozygous. No other polymorphisms at codon 198 were detected and there were no polymorphisms at codon 167 either.

## Discussion

Knowledge of the mechanisms contributing to AR can help inform strategies designed to control the development and spread of AR. While BZ resistance among ovine nematodes in Ireland is considered common [1–3], information regarding the polymorphisms associated with BZ resistance in Ireland has been lacking. Previous studies indicated *Teladorsagia* spp., *Cooperia* spp. and *Trichostrongylus* spp., as the predominant BZ resistance genera [1]. The results of this study are in agreement with these findings. *T. circumcincta* was identified as the most common BZ resistant species, being found on all four farms.

Molecular detection of anthelmintic resistance is desirable as it can provide a more sensitive method of detection than conventional methods such as the faecal egg count reduction test [11]. However, as molecular anthelmintic resistance detection methods are developed it is important they are validated for use in different populations. The most commonly reported indicator of BZ resistance in ovine nematodes is a T to A transition in the isotype 1 β-tubulin gene resulting in an F200Y substitution in the encoded protein. In agreement with previous studies, the F200Y substitution was evident in the majority (32/36) of BZ resistant *T. circumcincta* individuals in this study. It has been reported that this mutation is recessive in *T. circumcincta* and that only homozygous Y/Y individuals survive BZ treatment [19]. In agreement with this, all BZ surviving nematodes from two farms were homozygous for the mutation. However, on the Galway farm, in addition to the 7 nematodes homozygous for the mutation, one F/Y heterozygote was recovered. Interestingly, only one nematode from Wicklow was homozygous for the mutation with four heterozygous and four homozygous for the wild-type allele. A non-synonymous mutation at codon 198 (E198L) was also found in two worms from Wicklow. This E198L mutation has been reported to be associated with BZ resistance in *T. circumcincta* populations in the United Kingdom [20]. The amino acid at this position may be important in the mechanism of action of BZ as the E198A mutation has been experimentally shown to confer a greater level of resistance than the F200Y mutation in *H. contortus* [21]. However, the role of the E198L mutation in anthelmintic resistance remains to be definitely determined. In the Wicklow worm population, the frequency of the TAC polymorphism, found at amino acid position 200 and associated with resistance, was 0.33; indicating another BZ resistance mechanism may be present on this farm. Studies with *H. contortus* have indicated that the isotype-2 β-tubulin genes may also be of interest in terms of additional BZ resistance mechanisms [22]. Furthermore, other mechanisms of BZ resistance are suspected with the P-glycoproteins also implicated [23].

In agreement with other studies, the F200Y substitution resulting from a mutation in the isotype 1 β-tubulin coding gene was the most commonly observed mutation in BZ-resistant *T. circumcincta* in Ireland. In light of these findings, application of molecular tests to detect BZ resistance in *T. circumcincta* could prove useful, however, caution is warranted as molecular tests based on known substitutions have the potential to leave some instances of resistance undetected.

## Abbreviations

AR: Anthelmintic resistance
BZ: Benzimidazole
DAFM: Department of Agriculture Food and the Marine
EPG: Eggs per gram
FEC: Faecal egg count
GIN: Gastrointestinal nematode
PCR: Polymerase chain reaction
SNP: Single nucleotide polymorphism
